# Merger of *Betula tatewakiana* (Betulaceae) from northern Japan with northeast Asian *B. ovalifolia* based on ploidy level

**DOI:** 10.3897/phytokeys.170.58585

**Published:** 2020-12-23

**Authors:** Yuki Shiotani, Tomoko Fukuda, Elena A. Marchuk, Ekaterina A. Petrunenko, Pavel V. Krestov, Svetlana N. Bondarchuk, Yoko Nishikawa, Takashi Shimamura, Yoshiyasu Fujimura, Koh Nakamura

**Affiliations:** 1 Division of Biosphere Science, Graduate School of Environment Science, Hokkaido University, Sapporo 060-0810, Japan; 2 College of Liberal Arts and Sciences, Mie University, Tsu 514-8507, Japan; 3 Botanical Garden-Institute, Far Eastern Branch of the Russian Academy of Sciences, Makovskii Str. 142, Vladivostok, 690024, Russia; 4 Sikhote–Alin State Nature Biosphere Reserve, Ministry of Nature Resources and Environment, Terney, 692150, Russia; 5 Institute of Environmental Sciences, Hokkaido Research Organization, Sapporo 060-0819, Japan; 6 Research & Development Center, Nippon Koei Co. Ltd., Tsukuba 300-1259, Japan; 7 Botanic Garden, Field Science Center for Northern Biosphere, Hokkaido University, Sapporo 060-0003, Japan

**Keywords:** *
Betula
*, chromosome number, conservation, dwarf birch, endangered species, Hokkaido, Japan, polyploidy, Russian Far East, wetland

## Abstract

It has been controversial whether *Betula tatewakiana*, a dwarf birch distributed in Hokkaido of northern Japan, is an endemic species or a synonym of *B. ovalifolia* broadly distributed in northeast Asia. The endemic hypothesis is based on the idea that *B. tatewakiana* is diploid while *B. ovalifolia* is tetraploid and that they are separated based on the ploidy level; however, no chromosome data have actually been published before. Resolving the taxonomic problem is crucial also in judging the conservation priority of *B. tatewakiana* in a global perspective. Our chromosome observation revealed that *B. tatewakiana* is tetraploid as well as *B. ovalifolia*. We also conducted morphological observations and clarified that *B. tatewakiana* is morphologically identical to *B. ovalifolia* in white hairs and dense resinous glands respectively on adaxial and abaxial leaf surfaces, in which they differ from closely related species in the same section Fruticosae. We conclude that the hypothesis that *B. tatewakiana* is a Hokkaido endemic based on the ploidy level is not supported and that *B. tatewakiana* should be merged with *B. ovalifolia*.

## Introduction

**Figure 1.**
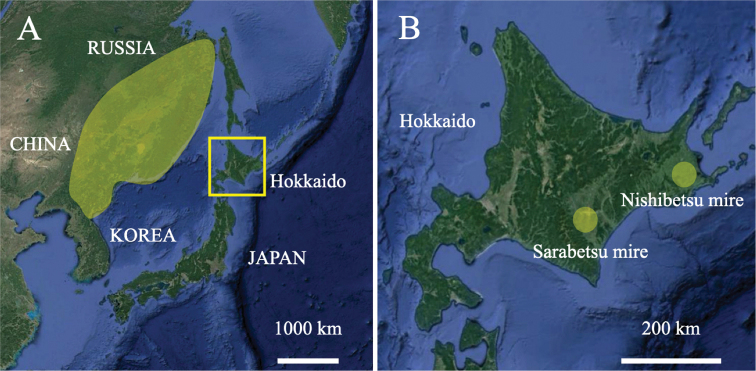
Species distribution ranges of *Betula ovalifolia* (**A**) and *B. tatewakiana* (**B**). Map data 2020 (C) Google.

*Betula ovalifolia* Rupr. is a dwarf birch found in wetlands (Gray 1996; Li and Skvortsov 1999). It grows only up to ca. 2 m tall (Li and Skvortsov 1999) and reproduces not only by seeds but also asexually by branching near ground level (Tabata 1966). This species is widely distributed in northeast Asia, i.e., Russian Far East (southern Khabarovsky Krai, Primorsky Krai, Amur Oblast, Jewish Autonomous Oblast), northeast China (Heilongjiang, Changbai Shan, Nei Mongol), North Korea, and northern Japan (Hokkaido) (Fig. 1; Gray 1996; Li and Skvortsov 1999). However, the taxonomic treatment of *B. ovalifolia* from Japan, and thereby its occurrence in Japan, has been controversial. In the first report from Japan, it was treated as an endemic species of Hokkaido and named *B. tatewakiana* M.Ohki & S.Watan. (Watanabe and Ohki 1959). Afterward, it had been treated as a variety of *B. humilis* Schrank (Murata 1978). Soon after that, Hara (1979) claimed that this species is identical to *B. ovalifolia*, but no data were presented. Since then, although this opinion is widely accepted in pictorial books and floras in Japan (Murata 1979; Ito 1981; Ohba 2006; Takahashi 2015), the idea to support *B. tatewakiana* was claimed again (Watanabe 1995) and the taxonomic problem still remains (Takahashi 2013; Takahashi et al. 2013; Nemoto 2016). Here, we tentatively use the name *B. tatewakiana* and will later discuss its taxonomy and proper name based on our results.

The taxonomic problem of *B. tatewakiana* and *B. ovalifolia* stems from the confusion in their ploidy level. Watanabe (1995) claimed that *B. tatewakiana* is diploid while *B. ovalifolia* is tetraploid and he recognized *B. tatewakiana* as the Japanese endemic restricted to Hokkaido. This idea, however, was originally reported in a conference abstract without images of the chromosomes (Watanabe and Somego 1991) and has never been published, but repeatedly mentioned in the following studies (Nemoto 2016). On the other hand, Nagamitsu et al. (2004) did not separate the two species and treated *B. ovalifolia* from Hokkaido as a tetraploid species based on Probatova and Sokolovskaya (1995, in Russian), who actually did not report the chromosome number of *B. ovalifolia* but of a hybrid between *B. ovalifolia* and *B. exilis*. A flow cytometric study of the genome size evolution in the genus *Betula* suggested that *B. ovalifolia* from the Asian continent is tetraploid (Wang et al. 2016). Ashburner and McAllister (2016) treated *B. tatewakiana* and *B. ovalifolia* as synonyms of *B. fruticosa* Pall. and reported the chromosome number 2*n* = 56 for *B. fruticosa*. According to the author (McAllister, personal communication), the chromosome observations were made with plants grown from seeds collected in Sarabetsu mire and Olga in Russian Far East. However, the materials were not mentioned in Ashburner and McAllister (2016) and one cannot be certain that the chromosome number applies to *B. tatewakiana* and *B. ovalifolia*. Thus, no published information exists about the ploidy level of *B. tatewakiana* and *B. ovalifolia* based on chromosome observations.

In this study, to resolve the taxonomic problem of *B. tatewakiana*, we focused on the confusion about the ploidy level, because this is the cause of the taxonomic controversy. We conducted chromosome observation and determined the ploidy level. We also conducted morphological observations of *B. tatewakiana*. Regarding *B. ovalifolia*, there are two closely related species in the same section Fruticosae, i.e., *B. humilis* Schrank and *B. fruticosa* Pall. *Betula ovalifolia* is distinguished from the two species by white hairs on the adaxial leaf surface (vs. glabrous in *B. humilis* and *B. fruticosa*) and by densely resinous glands on the abaxial leaf surface (vs. lack of glands in *B. humilis*) (Kuzeneva 1985, Li and Skvortsov 1999). In previous studies, which did not accept *B. tatewakiana*, these traits have not been well compared between *B. tatewakiana* and *B. ovalifolia*. Resolving the taxonomic problem and assessing the endemic status of *B. tatewakiana* would also help planning its conservation. *Betula tatewakiana* is found only in two localities in Japan, i.e., Sarabetsu and Nishibetsu mires in eastern Hokkaido (Fig. 1B). As a result of the exploitation of the mires, remaining habitats are only 3 and 16 ha in Sarabetsu and Nishibetsu mires, respectively (Takahashi 2013). Open ditches excavated inside and outside the mires are increasingly drying the habitats of *B. tatewakiana* and thereby it is red-listed at national and prefectural levels (Hokkaido 2001; Ministry of the Environment, Government of Japan 2020). Whether it is endemic or not is related to its conservation priority in a global perspective; on the other hand, if it is the same species as *B. ovalifolia* broadly distributed in northeast Asia, effective conservation should be planned considering genetic connectivity with conspecific populations abroad. This study is expected to provide basic information essential for the conservation of the species.

## Materials and methods

### Determination of ploidy level

We collected seeds of *B. tatewakiana* from six and five individuals from Sarabetsu and Nishibetsu mires in Hokkaido, Japan; seeds of *B. ovalifolia* were collected from one individual in Sikhote–Alin Nature Reserve in Primorsky Krai, Russian Far East (Table 1). Collected seeds were dried with silica gel and stored at 4 °C. Seeds were sowed on vermiculite and germinated at 25 °C day / 8 °C night condition for two weeks. After germination, root tips were collected and pretreated with 0.002 M 8-hydroxyquinoline solution for 24 hours at 4 °C in dark condition. Next, the root tips were fixed in Farmer’s solution (glacial acetic acid: 99% ethanol = 1: 3) at 4 °C in dark condition. After fixation, the root tips were macerated in 1 N HCl for 18 minutes and stained with 1% aceto-orcein for 5 minutes and squashed on a slide. Metaphase chromosomes were observed using an optical microscope Zeiss Axio Imager A1 (Carl Zeiss, Jena, Germany), and pictured by Anyty 3R-DKMC01 (3R solution corp., Fukuda, Japan).

**Table 1.** Chromosome counts of *Betula tatewakiana* and *B. ovalifolia*.

Taxon	Sampling site	Chromosome counts	Voucher no.
*B. tatewakiana*	42°37.33'N, 143°15.72'E, alt. 166 m, Sarabetsu mire, Sarabetsu village, Hokkaido, Japan	56	HUBG* 14746 A, E, H
		ca. 56	HUBG 14746 B, D, F
	43°23.36'N, 145°03.66'E, alt. 32 m, Nishibetsu mire, Betsukai town, Hokkaido, Japan	56	Yuki Shiotani 1, 26, 29, 30 (SAPT)
		ca. 56	Yuki Shiotani 27 (SAPT)
*B. ovalifolia*	44°57.31'N, 136°33.01'E, alt. 25 m, Sikhote–Alin Nature Reserve, Terney, Primorsky Krai, Russia	56	Koh Nakamura 14198 (SAPT)

*HUBG: living collections of Hokkaido University Botanic Garden.

### Morphological observations

To elucidate whether *B. tatewakiana* is morphologically identical to *B. ovalifolia* or not, we observed the key traits in the section Fruticosae: white hairs and dense resinous glands respectively on the adaxial and abaxial leaf surfaces. For *B. tatewakiana*, specimens examined were the holotype of *B. tatewakiana* (H. Suzuki and M. Ohki, s.n. with handwriting “Type” and collected on 18 August 1958 as cited in the protologue) in the herbarium of Hokkaido University Museum (SAPS) and our collections of 51 and 45 plants from Sarabetsu and Nishibetsu mires, that were deposited in the herbarium of Hokkaido University Botanic Garden (SAPT) (Appendix 1). For *B. ovalifolia*, our collections of 38 specimens from Primorsky Krai in the Russian Far East were used (SAPT, Appendix 1).

## Results

### Ploidy level

**Figure 2.**
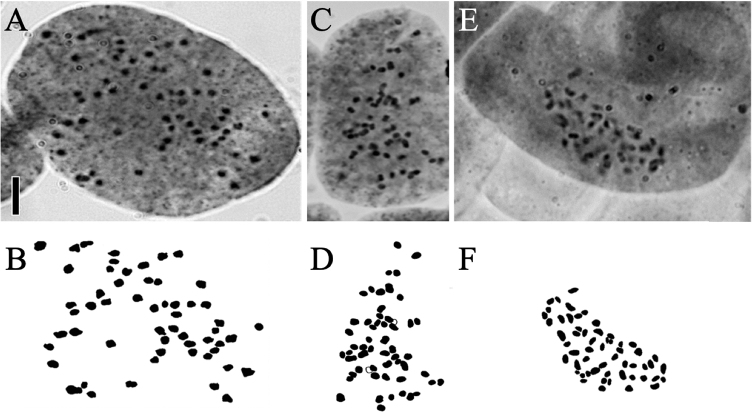
Somatic chromosomes at metaphase of *B. tatewakiana* and *B. ovalifolia*. Photomicrographs of *B. tatewakiana* from Sarabetsu mire (**A**, 2*n* = 56: HUBG 14746 A) and Nishibetsu mire (**C**, 2*n* = 56: Yuki Shiotani 29), and *B. ovalifolia* from Primorsky Krai (**E**, 2*n* = 56: Koh Nakamura 14198) are shown. **B, D, F** are drawings of **A, C, E**, respectively. Scale bar: 5 µm.

Somatic chromosomes at metaphase were approximately 1.0 µm long in both *B. tatewakiana* (Fig. 2A–D) and *B. ovalifolia* (Fig. 2E, F). The centromere positions could not be determined because of the small sizes of the chromosomes. The result of chromosome counts is summarized in Table 1. In *B. tatewakiana* from Sarabetsu mire, 3 individuals had 56 chromosomes (HUBG 14746 A, E, H) and 3 individuals had ca. 56 chromosomes (HUBG 14746 B, D, F). In *B. tatewakiana* from Nishibetsu mire, 4 individuals had 56 chromosomes (Yuki Shiotani 1, 26, 29, 30) and 1 individual had ca. 56 chromosomes (Yuki Shiotani 27). In *B. ovalifolia* from Primorsky Krai, 1 individual had 56 chromosomes (Koh Nakamura 14198).

### Morphological traits

**Figure 3.**
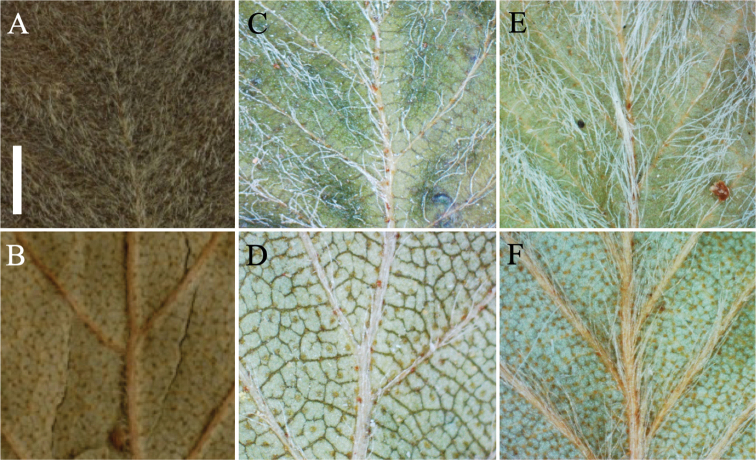
Leaf traits of *B. tatewakiana* and *B. ovalifolia*. White hairs on adaxial leaf surface (**A, C, E**) and densely resinous glands on abaxial leaf surface (**B, D, F**) are shown for the holotype of *B. tatewakiana* (H. Suzuki and M. Ohki, s.n., **A, B**), *B. tatewakiana* of our collection (Yuki Shiotani 38, **C, D**), and *B. ovalifolia* in Russia (Koh Nakamura 14188, **E, F**). Scale bar: 1 mm.

The holotype of *B. tatewakiana* has white hairs and dense resinous glands respectively on the adaxial and abaxial leaf surface (Fig. 3A, B). Our collections of *B. tatewakiana* also have white hairs and dense resinous glands on the adaxial and abaxial leaf surface, respectively (Fig. 3C, D) and no morphological difference was recognized between the samples from Sarabetsu and Nishibetsu mires. In *B. ovalifolia*, our collections from Primorsky Krai have white hairs and dense resinous glands respectively on adaxial and abaxial leaf surface as well as *B. tatewakiana* (Fig. 3E, F).

## Discussion

### Merger of *B. tatewakiana* with *B. ovalifolia*

In our chromosome observation, the samples of *B. tatewakiana* from Sarabetsu and Nishibetsu mires had 2*n* = 56 (seven samples) and ca. 56 (four samples) chromosomes (Table 1). The chromosomes were too small (approximately 1.0 µm long) to observe clearly and the chromosome count variation may need further verification; however, it is safe to say that *B. tatewakiana* is tetraploid because the basic chromosome number is 14 in the genus *Betula* (Erikkson and Jonsson 1986) and the diploid count should be 2*n* = 28. Watanabe and Somego (1991) reported that *B. tatewakiana* is diploid, although no images of the chromosomes were presented. Thus, the possibility that there are both diploid and tetraploid in *B. tatewakiana* is not totally denied. However, his report was a gametophytic count and according to the author Watanabe the chromosome image was unclear (personal communication). For this reason, *B. tatewakiana* is highly likely to be tetraploid. Our chromosome count of *B. ovalifolia* was 2*n* = 56. This is consistent with the flow cytometric study that suggested that *B. ovalifolia* from the Asian continent is tetraploid (Wang et al. 2016). Therefore, the idea to separate *B. tatewakiana* from *B. ovalifolia* based on the ploidy level (Watanabe and Somego 1991; Watanabe 1995) is not supported because both species are tetraploid. Hence, *B. tatewakiana* should be merged with *B. ovalifolia*. The observation of the morphological traits also supports the merger of *B. tatewakiana* with *B. ovalifolia*. The two species are morphologically identical in white hairs and dense resinous glands respectively on the adaxial and abaxial leaf surfaces, based on which they are different from closely related dwarf birch species in the same section Fruticosae.

### Taxonomic treatment

***Betula ovalifolia* Rupr., Bull. Cl. Phys.-Math. Acad. Imp. Sci. Saint-Pétersbourg 15: 378 (1857)**

≡ *B. tatewakiana* M.Ohki & S.Watan., J. Jap. Bot. 34: 329 (1959). Type: Japan, Hokkaido, Sarabetsu village: 18 August 1958, H. Suzuki and M. Ohki, s.n. (holotype, SAPS!)

**Type.** Russia. Khabarovsk region: Mandshuria, 25 July 1855, R. Maack, s.n. (holotype, LE 01016954!)

### Implications for conservation

*Betula tatewakiana* is recognized as a synonym of *B. ovalifolia* as discussed above, and thereby it is not a Japanese endemic species. Hereafter, the Hokkaido populations are called *B. ovalifolia*. Because *B. ovalifolia* is broadly distributed in northeast Asia, i.e., Russian Far East, northeast China, North Korea, and northern Japan, the conservation priority of the species may not be high in a global perspective. On the other hand, the Hokkaido populations represent the only island populations disjunct from continental populations. The species had likely moved southward during glacial periods and retreated northward in warmer periods, and the Hokkaido populations are considered to be relict populations (Takahashi 2013). The Hokkaido populations can be reproductively isolated from the continental populations and can have a unique gene pool that deserves conservation. Also, in Japan, *B. ovalifolia* is distributed only in Sarabetsu and Nishibetsu mires and deserves conservation as national resource. On the other hand, if there exists gene flow among Hokkaido and continental populations, effective conservation should be planned considering genetic connectivity with populations abroad. Population genetics of *B. ovalifolia* in northeast Asia for conservation is the topic of our future investigation.

## Appendix 1

Specimens for morphological observation:

***Betula tatewakiana***

JAPAN, Hokkaido: Sarabetsu village, 18 August 1958, (H. Suzuki and M. Ohki, s.n., holotype, SAPS); Sarabetsu mire, 42°37.33'N, 143°15.72'E, alt. 166 m, 5 June 2017, Yuki Shiotani 65–115 (51 specimens, SAPT); Nishibetsu mire, 43°23.36'N, 145°03.66'E, alt. 32 m, 7 June 2017, Yuki Shiotani 1–26, 31–49 (45 specimens, SAPT)

***B. ovalifolia***

RUSSIA, Primorsky Krai: Terney, 44°57.31'N, 136°33.01'E, alt. 25 m, 22 July 2016, Koh Nakamura 14169–14195, 14197, 14198 (29 specimens, SAPT); Terney, 44°56.85'N, 136°33.00'E, alt. 9 m, 23 July 2016, Koh Nakamura 14289–14297 (9 specimens, SAPT)
